# Solid-State ^2^H NMR Analysis for Hierarchical Water Clusters Confined to Quasi-One-Dimensional Molecular Nanoporous Crystals

**DOI:** 10.1021/jacs.5c04573

**Published:** 2025-08-27

**Authors:** Tomoya Namiki, Akira Saito, Fumiya Kobayashi, Takuya Kurihara, Motohiro Mizuno, Makoto Tadokoro

**Affiliations:** † Department of Chemistry, Faculty of Science, 26413 Tokyo University of Science, Kagurazaka 1-3, Shinjuku-ku, Tokyo 162-8601, Japan; ‡ Department of Chemistry, Graduate School of Natural Science and Technology, 12858 Kanazawa University, Kakuma-machi, Kanazawa, Ishikawa 920-1192, Japan; § Institute for Frontier Science Initiative, 12858 Kanazawa University, Kakuma-machi, Kanazawa, Ishikawa 920-1192, Japan

## Abstract

Water confined to the quasi-one-dimensional hydrophilic molecular nanoporous crystal of {[Co^II^(H_2_bim)_3_]­(TMA)·20H_2_O}_*n*_ (**1**) (H_2_bim = 2,2′-biimidazole, TMA^3–^ = trimesate) forms dynamic water molecule clusters (WMCs) with a hierarchical three-layered hydrogen-bonding (H-bonding) structure and a time-averaged structure in the melting state due to interactions with the walls of the ∼1.6 nm nanopores. This was first revealed by measuring the solid-state ^2^H NMR spectra of a single crystal of {[Co^III^(D_2_bim)_3_]­(TMA)·20D_2_O}_*n*_ (**1′**), which revealed distinct hierarchical peaks of H_2_O H-bonding interactions. In addition, the frozen WMCs in **1** exhibit a premelting state, retaining some ice structures, just before melting through a first-order phase transition during the heating process. Measurements of the spin–lattice relaxation time (*T*_1_) in solid-state ^2^H NMR revealed rapid rotation motions for water molecules in these premelting WMCs, resulting in a correlation time τ closer to that of bulk liquid water.

## Graphical abstract

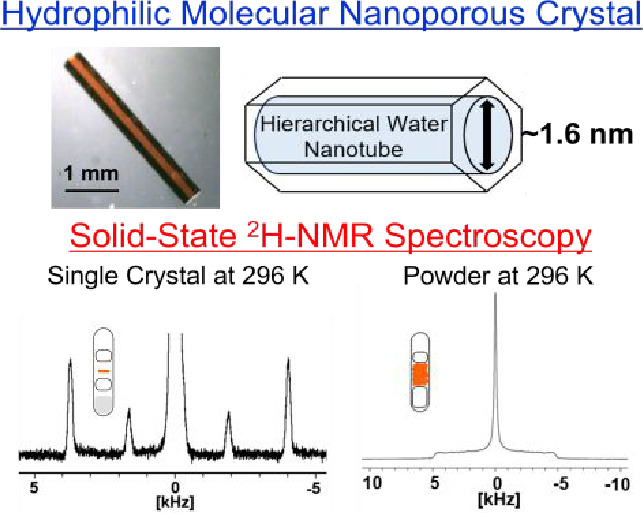

## Introduction

Water confined to nanopores exhibits unique physical and chemical properties that are distinct from those of bulk water owing to the effects of confinement in restricted nanospaces and interactions with pore interfaces.^1–5^ The unique properties of confined water play a crucial role in biological and hydrological processes.^6–14^ We previously reported on the properties of water molecule clusters (WMCs) confined to the hydrophilic nanoporous crystal {[Co^III^(H_2_bim)_3_]­(TMA)·20H_2_O}_*n*_ (**1**) (H_2_bim = 2,2′-biimidazole, TMA^3–^ = trimesate), which features quasi-one-dimensional (1-D) nanopores with a diameter of approximately 1.6 nm (Figure S1).^15–22^ H_2_O of the WMCs in **1** are stabilized by strong hydrogen bonds (H-bonds) with the O atoms of TMA^3–^ anions on the pore walls. The WMCs exist as dynamic clusters with a hierarchical three-layered structure of H-bonding interactions.^15^ In the tertiary region near the center of the WMCs, the H_2_O cannot form stable tetrahedral H-bonding structures and instead form incomplete H-bonding structures. Consequently, the H_2_O are highly mobile and resistant to freezing. During the freezing process of the WMCs, water starts freezing from the outer regions, where the H-bonding interactions are stronger, and it becomes increasingly difficult for H_2_O to freeze as freezing progresses toward the center.^18^ The hierarchical multilayered structure and freezing process of such WMCs are distinct not only from those of bulk water but also from the properties of WMCs confined to carbon nanotubes and mesoporous silica.^23–29^ Furthermore, WMCs that are frozen in the nanopores of **1** may exhibit a premelting state before undergoing melting through a first-order phase transition with increasing temperature.^19,22^ The premelting state involves the melting of incomplete H-bonded H_2_O before the completely frozen ice structure starts melting during the heating process. This suggests a novel phase of water in which frozen H_2_O layers and slowly moving H_2_O coexist.^22^

Solid-state ^2^H NMR spectroscopy is a highly effective technique for studying the dynamics of D_2_O in localized environments. Measuring spectra and relaxation times enables the investigation of the velocity and type of molecular motion, making ^2^H NMR spectroscopy a powerful tool for examining the hierarchical structure of D_2_O confined in nanopores.^30–36^ In this study, we first performed solid-state ^2^H NMR measurements at room temperature on single crystals of **1** containing dynamic WMCs confined to nanopores. Spectral peaks corresponding to the primary and secondary layers of the hierarchical three-layered structure are observed.

The crystal structure of the dynamic WMCs in the melting state confined to **1** could not be elucidated by X-ray structural analysis and neutron structural analysis (Figure S2).^19^ However, the dynamic WMC structure was first revealed through the X-ray structural analysis of {[Ru^III^(H_2_bim)_3_]­(TMA)·20H_2_O}_*n*_ (**2**), as shown in Figure 1a.^37^ The reason for the existence of a structuralized dynamic WMCs in the melting state of H_2_O is that, although individual H_2_O in the melting state are extremely fast moving (on the picosecond time scale), their motion becomes slow at locations where H-bonding interactions are temporarily concentrated. Owing to the confinement of water, a hierarchical three-layered structure, which is observable as the time-averaged electronic density of the O atoms in H_2_O, was revealed. This structure consists of a repeating unit of 20 H_2_O: the primary layer comprises a ring-like cluster formed from six H_2_O strongly H-bonded to O atoms of TMA^3–^ on the pore walls and other six H_2_O that are not directly H-bonded to O atoms; the secondary layer consists of a hexagonal cluster structure of six H_2_O; and in the tertiary region, two H_2_O are present near the center. The total outer H-bonding structure resembles that of a gas-hydrate with a 5^12^6^2^ cage.^38^ Given that the freezing structures of the premelting WMCs in **1** and **2** were similar, the structure of the dynamic WMCs in the melting state would also be similar.

**Figure 1:**
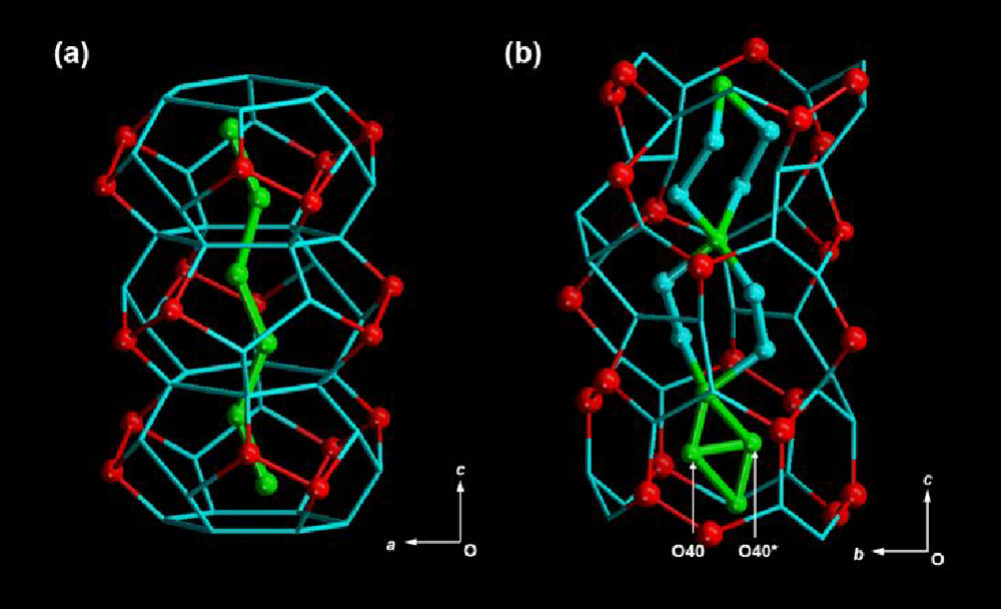
(a) Crystal structure of three units of WMCs in **2** at 253 K. The primary layers consist of six H_2_O H-bonded to O atoms of TMA^3–^ on the pore wall surface (red spheres and lines) and six H_2_O not directly H-bonded to O atoms on the wall surface (blue lines), the secondary layers (blue lines) consist of six H_2_O, and the tertiary regions (green spheres and lines) consist of two H_2_O. (b) Unit structure of the premelting state in crystal **1** at 198 K. The primary layer consists of 18 H_2_O H-bonded to the O atoms of TMA^3–^ on the wall surface (red spheres and lines) and 30 H_2_O not directly H-bonded to O atoms on the pore wall surface (blue lines), the secondary layer (blue spheres and lines) consists of eight H_2_O, and the tertiary region (green spheres and lines) consists of four H_2_O (O40 and O40* are disordered with an occupancy factor of 0.5).

The WMCs in **1** exhibited an endothermic peak at 245 K and exothermic one at 234 K due to a first-order melting and freezing phase transition, respectively, which was 28 K lower than that of bulk ice, as observed by differential scanning calorimetry (DSC) (Figure S3).^15,19,22^ In addition, a broad and weak endothermic peak due to premelting was observed between 210 and 240 K. Single-crystal X-ray structural analysis of **1** at 198 K, which retained the premelting state, revealed a hierarchical three-layered structure, as shown in Figure 1b. This structure consists of a repeating unit of 60 H_2_O: the primary layer contains 18 H_2_O strongly H-bonded to O atoms of TMA^3–^ on the pore walls and 30 H_2_O molecules that are not directly H-bonded to O atoms; the secondary layer includes eight H_2_O H-bonded to H_2_O in the primary layer; and the tertiary region contains four H_2_O near the center. One H_2_O disorder on sites O(40) or O(40)* with half occupancy each in the tertiary region adopts a unique two-coordinated H-bonding configuration and slowly moves to a stable position over 24 h at 165 K.^39^ In this study, we prepared {[Co^III^(D_2_bim)_3_]­(TMA)·20D_2_O}_*n*_ (**1′**) and aimed to elucidate the hierarchical structure of WMCs and the dynamics of a premelting state using solid-state ^2^H NMR measurements.

## Results and Discussion

To investigate the hierarchical structure of D_2_O confined to **1′**, static solid-state ^2^H NMR measurements with angular dependence were performed at 296 K using single crystals. The deuterated **1′** was prepared by soaking nondeuterated **1** in D_2_O for 1 h and repeating the process twice. As shown in Figures 2a and S4, the single crystal of **1′** was initially oriented perpendicular to the magnetic field *B*_0_ such that the long direction of the sample tube aligned with the *c*-axis channel direction of **1′**, and the ^2^H NMR spectra were recorded while rotating the sample tube in increments of θ = 15° from 0° to 360° in the *ab*-plane direction. Similarly, in Figures 2b and S5, the *c*-axis channel direction of **1′** was aligned parallel to *B*_0_ at 0°, and the crystal was rotated about the *ab*-plane. In both cases, the ^2^H NMR spectra revealed two types of doublet peaks, AA′ and BB′, in the range of ±5 kHz, attributed to the orientation of D_2_O. The splitting widths (Δν_s_) of AA′ and BB′ varied with the rotation angle θ. This change in peak positions is due to the anisotropy of quadrupole interactions with the magnetic field. When the single crystal was rotated with the *c*-axis channel direction perpendicular to *B*_0_, Δν_s_ for AA′ and BB′ varied in-phase with θ, as shown in Figure 2c. In contrast, when the channel direction started to align parallel with *B*_0_, Δν_s_ for AA′ and BB′ was out-of-phase with θ, as shown in Figure 2d, with the phase of AA′ shifted by θ = 45°. Figure 2e shows the powder pattern of the solid-state ^2^H NMR spectrum of **1′** at 296 K. Four peaks of AA′ and BB′ were observed with angle dependence between ±5 kHz widths. However, in the powder pattern, a slight increase in intensity was confirmed as a flat peak pattern within the range of ±5 kHz. Thus, in the single-crystal pattern, there are two peaks with different orientational dependencies, that is, two types of D_2_O with different anisotropic mobilities.

**Figure 2:**
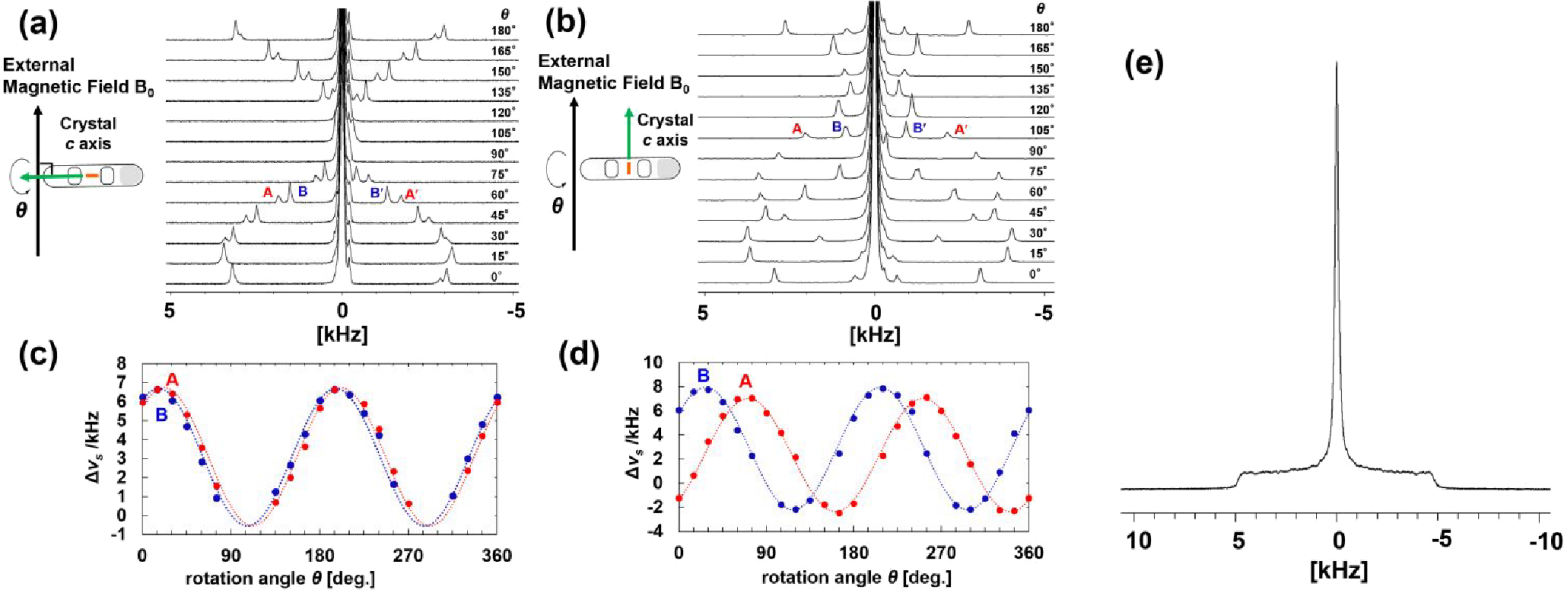
Solid-state ^2^H NMR spectra of a single crystal of **1′** at 296 K (θ = 0°–180°). (a) Long axis of the sample tube parallel to the *c* axis of the crystal (perpendicular to *B*_0_). (b) Long axis of the sample tube perpendicular to the *c* axis of the crystal (parallel to *B*_0_). (c, d) Angular dependence of peak splitting widths of the Δν_s_ of AA′ and BB′ in the solid-state ^2^H NMR spectra of a single crystal of **1′** perpendicular (c) and parallel (d) to *B*_0_. (e) Powder pattern of a solid-state ^2^H NMR spectrum of a powder sample of **1′** at 296 K.

Based on the dynamic WMCs structure in **2**, both the existence of two distinct peak positions for D_2_O and the 1:2 integral ratio suggest that AA′ corresponds to six D_2_O in the primary layer directly H-bonded to O atoms of TMA^3–^ on the pore walls, whereas BB′ corresponds to the six D_2_O in the primary layer not H-bonded to O atoms of TMA^3–^ and the six D_2_O in the secondary layer (Figure S6). The two D_2_O in the tertiary region, which exhibits very rapid isotropic rotation without peak splitting, overlaps at the spectral center with the peak of bulk D_2_O added for stabilization. These variations in peak positions indicate that there are hierarchical D_2_O structures with different H-bonding interactions in the WMCs at 296 K.

Static solid-state ^2^H NMR spectra of a crystalline powder sample of **1′** were measured during heating from 163 to 273 at 10 K intervals (Figure 3). At 163 K, a broad peak in the range of ±100 kHz was observed, indicating the frozen state of D_2_O in WMCs. However, the perfect Pake doublet structure was not observed, suggesting that D_2_O was not in a completely frozen state. The outer doublet in the range of Δν_p_ = ±75 kHz at 163 K disappeared at 193 K. Between 203 and 213 K, the broad peak sharply transitioned to a narrower peak in the range of ±5 kHz. This indicated that D_2_O in the pores partially melted, significantly increasing the mobility of D_2_O. In this temperature region, a phase transition from the frozen state to the premelting state, as observed by DSC, occurred. Furthermore, a distinct change in the line shape was observed between 243 and 253 K, corresponding to the first-order phase transition of melting (*T*_m_ = 250 K) of **1′** measured by DSC.^15^ The significant change in the line shape of the ^2^H NMR spectra from the frozen state to the premelting state suggests that D_2_O rotational motions considerably increased owing to the premelting phase transition. This cannot be rationalized only from the results of the X-ray structural analysis at 198 K, where the positions of the O atoms in H_2_O were nearly fixed, similar to those in the frozen state. Based on the X-ray structural analysis results, we fitted two static ^2^H NMR spectra at 163 and 263 K in the frozen and melting states, respectively, using the differences in the hierarchical structure of D_2_O (Figures S7 and S8). The fitting results are shown in Table 1, where the parameters are a quadrupole coupling constant (*C*_Q_), asymmetry factor (η), and exchange rate (*k*).

**Figure 3:**
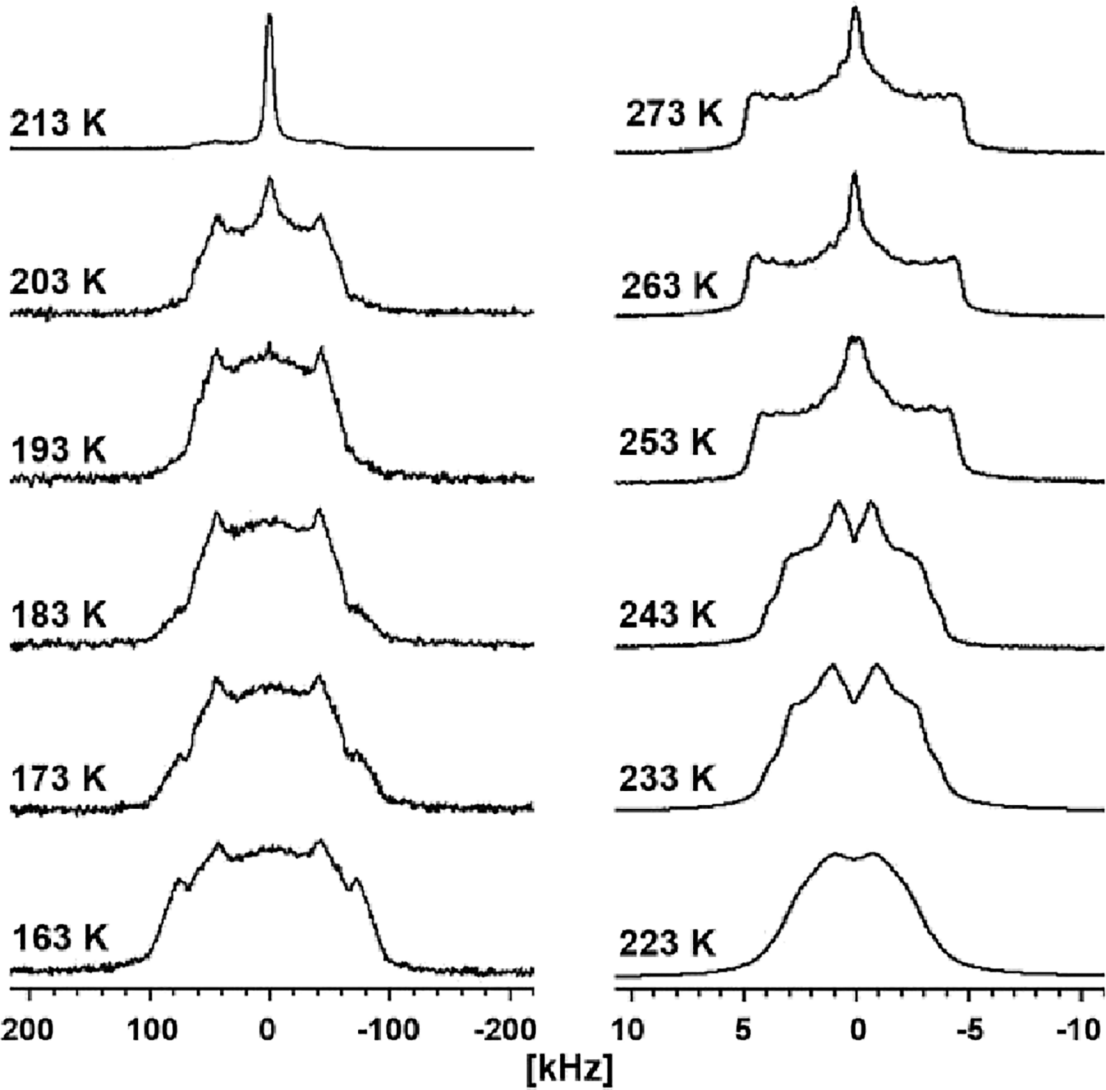
Static solid-state ^2^H NMR spectra for a powder sample of **1′** upon heating from 163 to 273 K in 10 K intervals.

**Table 1:** Parameter Values Used for the Fitting of Solid-State ^2^H-NMR Spectra for a Powder Sample of **1′** at 163 and 263 K^a^

*T* [K]	163			263			
component	1	2	3	1		2	3
molecular motion	rotational fluctuation (θ = 12°)	rotational fluctuation (θ = 44°)	isotropic rotation	restrained state + isotropic rotation	180° flip-flop + isotropic rotation	4-site jump	isotropic rotation
*C*_Q_ [kHz]	215	215	—	12^c^	12^c^	215	—
η	0.05	0.05	—	0.05	0.05	0.10	—
*k* [Hz]	1.0 × 10^8^	1.0 × 10^8^	—	—	1.0 × 10^8^	1.0 × 10^9^	—
fwhm [kHz]	—	—	50	—	—	—	0.70
integral ratio	4.5	9.5	1	3		6	1
no. of H_2_O^b^	18	38	4	6		12	2

a The hyphens “—” made no sense.

b The number of H_2_O in the repeating units was different between 163 and 263 K. The former was constructed from 60 H_2_O, and the latter was 20 H_2_O.

c This is the apparent *C*_Q_ = 12 kHz value.

Figure 4a shows the fitting results at 163 K for the frozen state. Based on the X-ray structural analysis, the frozen state of the WMCs consisted of a repeating unit of 60 D_2_O in a three-layered structure (Figure S9). When each peak was deconvoluted, component 1 (blue line) was assigned to 18 frozen D_2_O in the primary layer, that are strongly H-bonded to the O atoms of −COO^–^ groups on the pore walls. These data were fitted as a rotational fluctuation with θ = 12° motions (*C*_Q_ = 215 kHz, *k* = 1.0 × 10^8^ Hz, η = 0.05). Component 2 (green line) corresponds to 30 D_2_O in the primary layer that are not directly H-bonded to O atoms in the pore wall, combined with the eight D_2_O more loosely bound D_2_O in the secondary layer, totaling 38 D_2_O. These data were fitted by including a rotational fluctuation with θ = 44° motions (*C*_Q_ = 215 kHz, *k* = 1.0 × 10^8^ Hz, η = 0.05). Component 3 (yellow line) represents four D_2_O in the tertiary region, which is considered to exhibit an isotropic rotational motion and was fitted using a Lorentzian function, because the D_2_O are randomly involved in H-bonds around the center of WMCs. The sum of all peak components 1–3 (red line) closely matches the experimental peak (black line).

**Figure 4:**
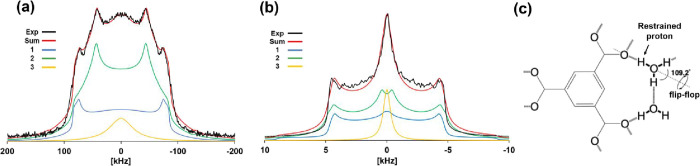
(a) Fitting of the ^2^H NMR spectrum of powder **1′** in the frozen state at 163 K. Component 1 (blue line) represents 18 D_2_O in the primary layer H-bonded to O atoms of −COO^–^ groups. Component 2 (green line) includes 30 D_2_O in the primary layer not H-bonded to O atoms on the pore wall and the eight frozen D_2_O in the secondary layer. Component 3 (yellow line) represents the isotopically moving four D_2_O in the tertiary region. The red line is the sum of components 1–3, and the black line represents the experimental data. (b) Fitting of the ^2^H NMR spectrum of powder pattern of **1′** in the melting state at 263 K. Component 1 (blue line) represents six D_2_O in the primary layer strongly H-bonded to O atoms of −COO^–^ groups in the pore wall. Component 2 denotes six D_2_O in the primary layer not directly H-bonded to O atoms on the pore wall, and six D_2_O in the secondary layer (green line), while component 3 represents the two isotopically moving D_2_O in the tertiary region (yellow line). The red line is the sum of components 1–3, and the black line represents the experimental data. (c) schematic representation is shown for the crystal structure of a cyclic H_2_O dimer strongly H-bonded to two O atoms of −COO^–^ groups as the primary layer, obtained by neutron structure analysis. The dimer exhibits a 180° flip-flop motion at an angle of 109.2° about the H-bonds to the O atoms, in addition to undergoing free rotational motion.

Figure 4b shows the fitting results at 263 K for the melting state. The fitting was also represented by three peak components. The repeating units of the WMCs in the melting state of **2**, as determined by X-ray crystal structure analysis, consisted of 20 D_2_O (Figure S10). The solid-state ^2^H NMR peak at 263 K can be divided into a three-layered structure of 20 D_2_O. It also revealed that six of these D_2_O belonged to the primary layer, which was strongly H-bonded to the O atoms of the −COO^–^ groups of the pore walls. Component 1 (blue line) corresponds to the D_2_O in the primary layer; during fitting, we used the overlapped line-shape that takes both the apparent *C*_Q_ = 12 kHz and a flip-flop motion into account. The former is a line-shape of D_2_O rotation and indicates that the small apparent *C*_Q_ value reduces the quadrupolar interaction due to the rotation of an entire D_2_O. The latter is interpreted as a 180° flip-flop motion of D_2_O rotating around an axis restrained by the O···D–O H-bond (the angle between the rotation axis and O···D is 54.6° (=109.2°/2). Figure 4c shows a schematic of the structure of strongly H-bonded H_2_O in a primary layer, obtained by neutron single crystal analysis at 298 K for **1** (Figure S11).^19^ 2 H_2_O H-bonded to O atoms of −COO^–^ groups form a H-bonding dimer structure with each other, and the whole H-bonding structure is stabilized by forming ring-shaped H-bonds. The neutron structure analysis also revealed a 180° flip-flop motion around O···H–O H-bonds between O atoms of −COO^–^ groups and H_2_O. Component 2 (green line) corresponds to six D_2_O in the secondary layer, and 6 D_2_O in the primary layer directly not H-bonded to O atoms on the pore wall totaling 12 D_2_O, which exhibit a 4-site jump motion (*C*_Q_ = 215 kHz, *k* = 1.0 × 10^9^ Hz, η = 0.10). The η value increased to 0.10 to account for a component of the rotational vibration.^40^ The two residual D_2_O exhibiting isotropic motion in the tertiary region of the WMCs were fitted using a Lorentzian function (yellow line). The sum of components 1–3 (red line) closely matches the experimental peak (black line).

To investigate the dynamics of D_2_O in confined WMCs, variable-temperature static ^2^H NMR spin–lattice relaxation time (*T*_1_) measurements (the resonance frequency of 61.44 MHz) were performed using the inversion recovery method on D_2_O-substituted powder **1′**. *T*_1_ measurements were performed by combining three components. Figure 5a shows the results obtained over a temperature range from 143 to 293 K, measured in 5 K intervals during the heating process, with measurements taken every 2.5 K in the range from 208 to 248 K to clearly observe the minimum values.

**Figure 5:**
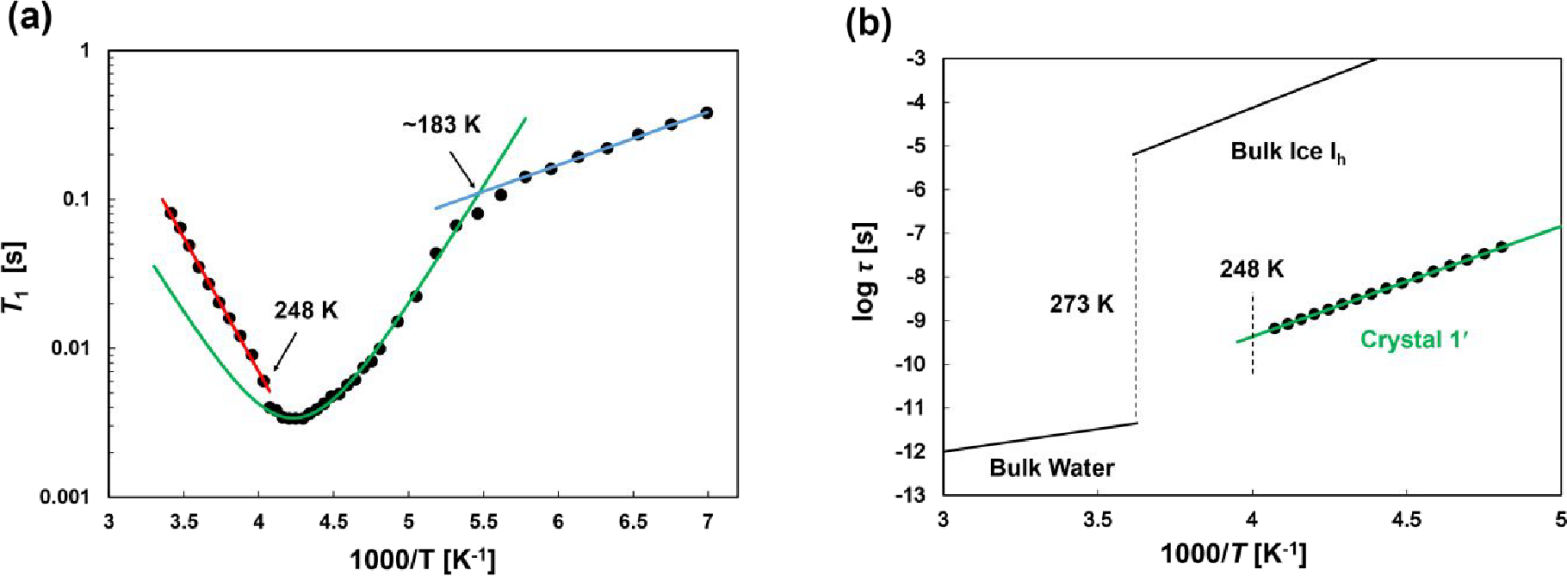
(a) Variable-temperature ^2^H spin–lattice relaxation time (*T*_1_) measurements from 143 to 293 K. Fitting was performed using Cole–Cole model (green line) and Arrhenius equations (red and blue lines). (b) Temperature dependence of correlation time τ for crystal **1′** compared with bulk water^50^ and bulk ice I_h._ ^49^

The *T*_1_ measurements revealed characteristic changes at approximately 183 K, corresponding to the transition from a frozen state to a premelting state, and at approximately 248 K, corresponding to the first-order phase transition of melting. Therefore, the *T*_1_ values of powder **1′** were divided into three temperature regions. From the ^2^H NMR measurements, the change in *T*_1_ at approximately 183 K marked the transition from a frozen state to a premelting state. Notably, the premelting state is observed from 183 to 243 K by DSC. Although most D_2_O are fixed in X-ray crystallography, the *T*_1_ measurements clearly indicate that D_2_O are actively undergoing rotational motion. The significant change from a curve to a straight line at approximately 248 K is attributed to a shift in the rotational motion and velocity of D_2_O, corresponding to the transition from the premelting state to a supercooled water region at the melting point.

The activation energies (*E*_a_) were estimated for the three temperature regions. Using the Arrhenius equation, the *E*_a_ values for the two linear ranges from 248 to 293 K and from 143 to 178 K were calculated to be 34 and 6.8 kJ/mol, respectively. In contrast, the temperature change in *T*_1_ from 183 to 243 K in the premelting region was exhibited a local minimum. Therefore, fitting was performed using the Cole–Cole model,^41,42^ an extension of the Bloembergen–Purcell–Pound (BPP) model because WMC confined to the nanopores of **1′** exhibits some molecular motions, such as D_2_O strongly held in place by H-bonds with the pore walls and more mobile ones near the center, leading to a distribution of correlation times, resulting in an *E*_a_ value of 48 kJ/mol. The coefficients of the BPP model depend on anisotropic molecular motion. The powder spectra of **1′** are composed of some anisotropic motions. Therefore, we have fitted it as a coefficient *C* = 1.39 × 10^11^ because of the residual anisotropy and calculated the *E*_a_ value. The high *E*_a_ value of 48 kJ/mol for the temperature range from 183 to 243 K is due to D_2_O being more stationary in premelting state, leading to a more developed H-bonding network. As the temperature increases, in the high-temperature range of the melting state from 248 to 293 K, a phase transition causes D_2_O to become more mobile, diminishing the H-bonding network. The *E*_a_ value of 34 kJ/mol in this range is close to the 24–35 kJ/mol observed for the isotropic rotational motion of D_2_O, indicating that it is similar to that of liquid water.^43^

The *E*_a_ value of dynamic WMCs in the premelting state on **1′** is larger than that of D_2_O in the supercooled water region. This is believed to be because those in the premelting state are more structuralized than those in the supercooled water region and have stronger H-bonding interactions. As a result, the larger *E*_a_ value in the premelting state is explained as differences of a stronger H-bonding interactions due to the structuration of D_2_O in the pores. In the temperature range from 143 to 188 K, the *E*_a_ value is lower at 6.8 kJ/mol. The lower *E*_a_ value is suggested to result from the vibrations and fluctuations of D_2_O in well-developed H-bonding networks, such as those in ice.^43^

Here, we discuss the effects on water confined to the crystalline molecular nanoporous material **1′** with 1-D regular hydrophilic walls. It is important to compare these results with those for SBA-15, a mesoporous silica with 1-D amorphous hydrophilic walls, and single-walled carbon nanotubes (SWCNTs), with 1-D hydrophobic walls. SBA-15 (with channels ∼5.4 nm in diameter) showed an *E*_a_ value of 89 kJ/mol,^44^ which is higher than that for **1′** in the supercooled water region (34 kJ/mol) and the premelting state (48 kJ/mol). In SBA-15, D_2_O easily binds to irregular −SiOSi– and −Si­(OH)­Si– groups on the channel walls, forming an unstable H-bonding network and exists as glass-forming liquids in the frozen state. The *E*_a_ value rapidly increases when Arrhenius-type fitting is performed for glass-forming liquids. Because the anomalous *E*_a_ value is due to a phenomenon that in the glass-forming liquid state D_2_O do not easily rapidly rotate as the temperature decreases by an amorphous state without a regular structure.^45^ SWCNTs (with channels 1.69–4.07 nm in diameter) has an *E*_a_ value 21–24 kJ/mol,^46^ which is lower than that for **1′** in the supercooled water region and premelting state. In SWCNTs, the van der Waals interactions between the aromatic regions of the walls and D_2_O are much weaker than the H-bonding interactions during the confined D_2_O, leading to the more developed H-bonding network among D_2_O close to the center of the pores.^46^ Therefore, the *E*_a_ value is closer to that of 22 kJ/mol^47^ for the H-bonding interactions of bulk water. In contrast, the D_2_O in crystalline **1′** forms a dynamic WMCs that is different from bulk liquid water because of H-bonds with the regularly positioned −COO^–^ groups on the pore walls. This structuralized D_2_O position results in a higher *E*_a_ value than that of SWCNTs. In previous studies, although no changes associated with phase transitions (i.e., collective motions of H_2_O molecules) were observed, crystallization water confined to the molecular crystal [Co^III^(en)_3_]­Cl_3_·4H_2_O, which possesses nanopores (∼0.44 nm), was found to reflect local dynamics and was investigated using a solid-state NMR measurement.^48^ The dynamics of this confined H_2_O were measured via the temperature-dependent line-width (*T*_2_ relaxation) of ^1^H MAS NMR, revealing an activation energy of *E*_a_ = 18 kJ/mol. In contrast, our crystal **1′** features larger crystalline channels with nanopores (∼1.6 nm), which have a phase transition and thus more developed intermolecular H-bonds, including collective motion of D_2_O, leading to a higher *E*_a_ value for D_2_O movement. In addition, although D_2_O easily move at this premelting state, the *E*_a_ value of 48 kJ/mol in the premelting state of **1′** is close to that of bulk ice (56 kJ/mol).^49^ However, D_2_O was found to have a very small correlation time on the order of τ ≈ 10^–9^ s at 243 K and high mobility, which are comparable to correlation times on that in MCM-41 from 230 to 245 K.^30^ (Figure 5b) This suggests that the positions of D_2_O in the premelting state are less mobile, and the O atoms of D_2_O molecules are relatively fixed. However, based on the correlation times and in terms of rotational D_2_O mobility, the D_2_O molecules are more mobile than in ice and closer to the state of liquid water.^50^

## Conclusions

Using static solid-state ^2^H NMR techniques, we elucidated the dynamics and hierarchy of the WMCs confined to quasi-1-D hydrophilic crystal pores for the first time. Solid-state ^2^H NMR measurements of a single crystal of **1′** revealed two distinct anisotropic peaks for the WMCs at room temperature. One peak corresponded to D_2_O in the primary layer that are H-bonded to the O atoms of the −COO^–^ groups on the pore wall. The other peak represented D_2_O in the primary layer that are not H-bonded to the O atoms of the −COO^–^ groups on the pore wall and the secondary layer.

The variable-temperature ^2^H NMR spectra reflected the mobility of D_2_O in the pores, with characteristic changes in their motion modes, exchange rates, and structural changes, indicating hierarchical and dynamic WMCs. Spectral fitting to the peaks corresponding to the frozen state at 163 K and supercooled water region at 263 K was successful with three hierarchical components, based on the WMCs structures derived from X-ray structural analysis. Variable-temperature measurements of the *T*_1_ values provided estimates of the *E*_a_ values across the three temperature regions, reflecting the changes in D_2_O mobility. The *E*_a_ value of 48 kJ/mol of the confined D_2_O in the premelting state of **1′** is close to that at 56 kJ/mol of bulk ice, indicating that similar to ice, it is difficult for D_2_O to move in this temperature range because it requires the disconnection of H-bonding interactions. However, the correlation time τ ≈ 10^–9^ s for **1′** at 243 K, which indicates that the mobility of D_2_O is closer to that of bulk liquid water at 273 K (τ ≈ 10^–11^ s), and far from that of bulk ice at 273 K (τ ≈ 10^–5^ s).

## Supplementary Material



## Notes

Experimental details, sample preparations, measurement conditions of powder and single crystals of **1′** on the solid-state ^2^H NMR spectra, DSC measurements, and the solid-state ^2^H NMR spectra for powder fitting conditions based on X-ray and Neutron structural analyses (PDF)
