# Supplementary material for: Solid-State 2H NMR Analysis for Hierarchical Water Clusters Confined to Quasi-One-Dimensional Molecular Nanoporous Crystals
Source: J Am Chem Soc. 2025 Aug 27;147(36):32440–6. doi: 10.1021/jacs.5c04573 (PMC12426917; doi:10.1021/jacs.5c04573)
Supplement: Supplementary file 1 [file ja5c04573_si_001.pdf]

Supporting Information for

Solid-State ^{2}H-NMR Analysis for Hierarchical Water Clusters Confined to *Quasi*-One-Dimensional Molecular Nanoporous Crystals

Tomoya Namiki,[a] Akira Saito,[a] Fumiya Kobayashi,[a] Takuya Kurihara,[b] Motohiro Mizuno,[b,c,d] Makoto Tadokoro*[a]

[a]Department of Chemistry, Faculty of Science, Tokyo University of Science, Kagurazaka 1-3, Shinjuku-ku, Tokyo 162-8601, Japan

[b]Department of Chemistry, Graduate School of Natural Science and Technology, Kanazawa University, Kakuma-machi 920-1192, Kanazawa, Ishikawa, Japan

[c]Nanomaterials Research Institute, Kanazawa University, Kakuma-machi 920-1192, Kanazawa, Ishikawa, Japan

[d]Institute for Frontier Science Initiative, Kanazawa University, Kakuma-machi 920-1192, Kanazawa, Ishikawa, Japan

e-mail: tadokoro@rs.tus.ac.jp

EXPERIMENTAL

Sample preparation

Synthesis of {[Co^{III}(H_2bim)$_3$](TMA)·20H_2O}$_n$ (**1**)

Crystal **1** was prepared according to the literature, except that [Co^{III}(H_2bim)$_3$](NO_3)$_3$ was used instead of [Ru^{III}(H_2bim)$_3$](NO_3)$_3$.[1] [Co^{III}(H_2bim)$_3$](NO_3)$_3$ (0.013 g, 0.02 mmol) and K_3TMA (0.007 g, 0.02 mmol) were slowly diffused in H_2O (10 cm^3) to give orange crystals with a hexagonal prism of **1** after three weeks.

Preparation of {[Co^{III}(D_2bim)$_3$](TMA)·20D_2O}$_n$ (**1′**)

Deuterated crystal **1′** was prepared by soaking non-deuterated crystal **1** in D_2O for 1 h, and repeating the process twice.

Preparation of Solid-State ^{2}H-NMR Measurement Samples

Approximately 20 mg of the crystalline powder sample or single crystals of **1′** was added to a hard glass tube (Φ ~4 mm), with the crystal position adjusted using a Teflon tape. To prevent the escape of D_2O from the crystal during the measurement process, the bottom of the sample tube was packed with cotton moistened with D_2O. The crystals were then frozen by immersion in liquid nitrogen. The glass tube was evacuated using a vacuum pump and sealed by flame cutting to prepare the sample for ^{2}H-NMR measurements.

Solid-State ^{2}H-NMR Spectra Measurements

Single-crystal and powder solid-state ^{2}H-NMR measurements were performed using a Bruker AVANCE NEO 400 spectrometer at resonance frequency of 61.44 MHz. The measurements were conducted using the quadrupole echo method ($90°_x$ – τ_1 – $90°_y$ – τ_2 – acq), 90° pulse width of 5 μs, recycle delay of 1.0 s, an and inter-pulse delay of τ_1 = 60 μs and τ_2 = 53 μs. Simulations of the ^{2}H-NMR spectra were performed using homemade Fortran programs with double precision.[2] The fitting parameters were used a 90˚ pulse width of 5 μs, and pulse intervals of τ_1 = 60 μs and τ_2 = 53 μs. The simulation of the rotational-vibration mode was conducted using a two-site flip model of deuteron Euler angles (α_1, β_1, γ_1) = (0, 0, 0), (α_2, β_2, γ_2) = (0, 12, 0) and (α_1, β_1, γ_1) = (0, 0, 0), (α_2, β_2, γ_2) = (0, 44, 0).

^{2}H Spin-Lattice Relaxation Time (T_1) Measurements

^{2}H spin-lattice relaxation time (T_1) measurements were performed using the inversion recovery method combined with a quadrupole echo method ($180°_x$ – t – $90°_x$ – τ_1 – $90°_y$ – τ_2 – acq), 180° pulse width of 10 μs, 90° pulse width of 5 μs, recycle delay of 1.0 s, and an inter-pulse delay of τ_1 = 60 μs and τ_2 = 53 μs. These spectra with 15–20 different delay times t

were used to determine the T_1 value at each temperature. The BPP equation used in the fitting is as follows:

$$T_1{}^{-1} = \frac{3\pi^2}{10}\left(\frac{e^2qQ}{h}\right)^2\left(1+\frac{\eta^2}{3}\right)[J(\omega)+4J(2\omega)]$$

where, ω, e^2qQ/h, and η are the resonance frequency, a quadrupole coupling constant, and an asymmetric parameter, respectively. However, the water confined to crystal **1'** have some anisotropic rotational motions that affect the relaxation, therefore, the coefficient was replaced as *C* because of the residual anisotropy.

$$T_1{}^{-1} = C[J(\omega)+4J(2\omega)]$$

We obtained C = 1.39 × 10^{11} and $J(\omega)$ was calculated using

$$J(\omega) = \frac{\tau_c}{1+(\omega\tau_c)^2}$$

where, $\boldsymbol{\tau}$ is correlation time. Additionally, when a distribution of correlation time exists, $J(\omega)$ is well described by the Cole–Cole model as follows:

$$J_{CC}(\omega) = \frac{\omega^{-1} sin\left(\frac{\pi}{2}\beta_{CC}\right)(\omega\tau_c)^{\beta_{CC}}}{1+(\omega\tau_c)^{2\beta_{CC}}+2cos\left(\frac{\pi}{2}\beta_{CC}\right)(\omega\tau_c)^{\beta_{CC}}}$$

The β_{CC} value takes a value in the range of 0–1. When the β_{CC} value is 1, the $J_{CC}(\omega)$ equation is equal to the BPP equation. We obtained β_{CC} = 0.64. To obtain τ_c, the following Arrhenius equation was used:

$$\tau_c = \tau_0 exp\left(\frac{E_a}{RT}\right)$$

We obtained τ_0 = 3.15 × 10^{-20} s.

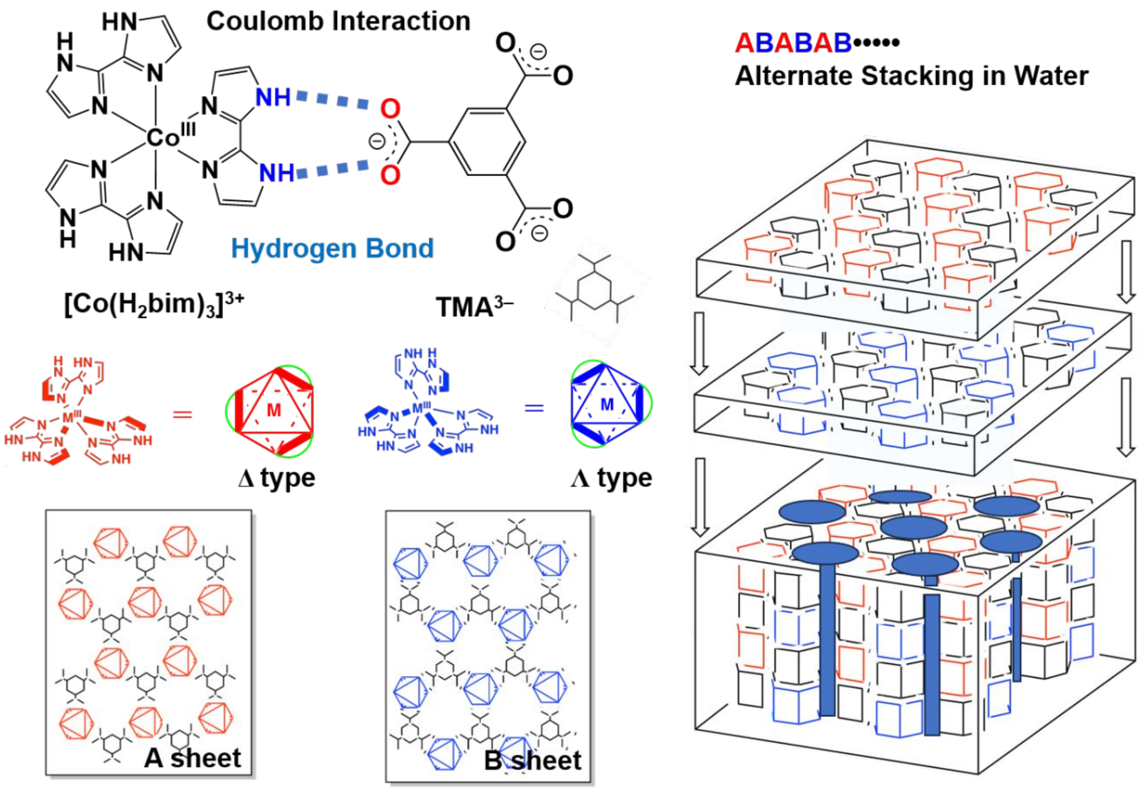

Figure S1. Synthetic structure of quasi-one-dimensional hydrophilic molecular nanoporous crystal. $[Co(H_2bim)_3]^{3+}$ and TMA^{3-} were formed by charge-assisted hydrogen bonds. The Co complex exists in Δ and Λ isomers, which form different two-dimensional honeycomb sheets (A sheet and B sheet), respectively. By stacking alternately, water molecular clusters were confined to their nanoporous crystal.

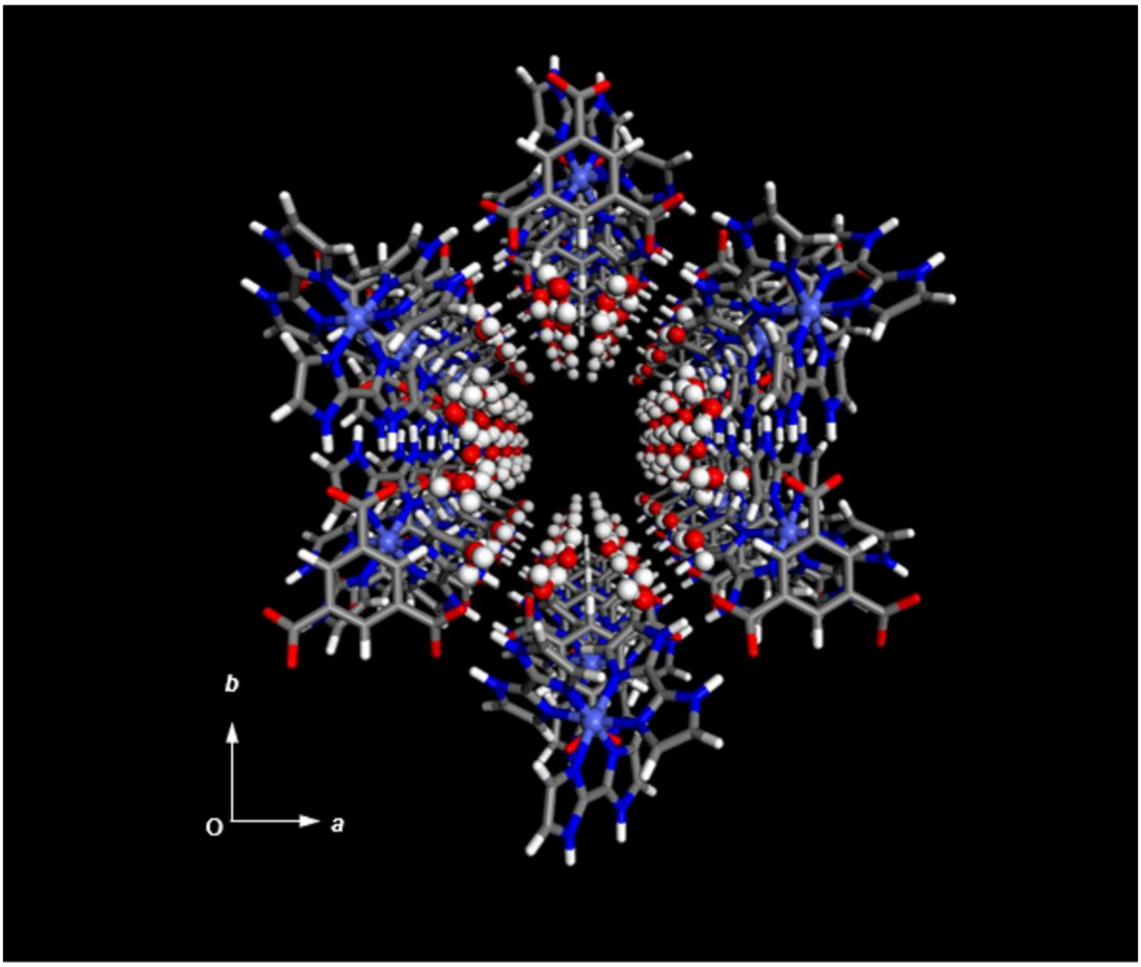

Figure S2. Neutron structural analysis of **1** at 293 K.[3] Color code: white, H; red, O; grey, C; blue, N; light blue, Co.

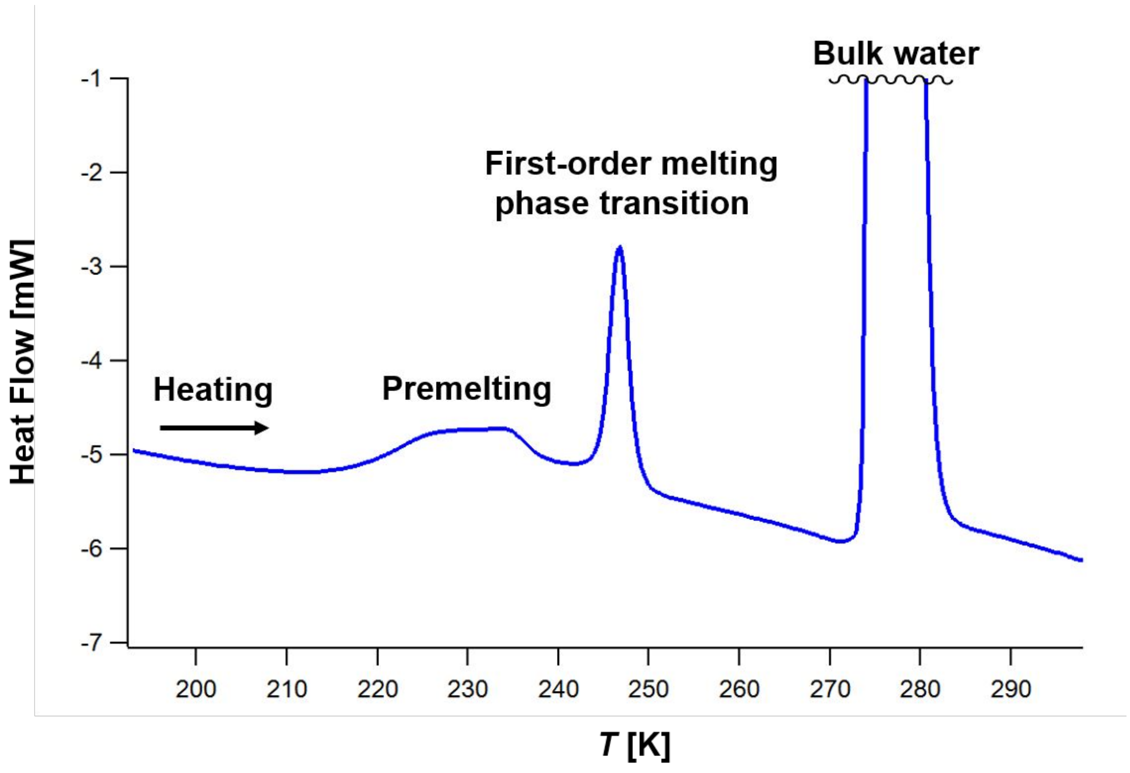

Figure S3. Differential scanning calorimetry thermogram of **1** was measured at a velocity of 5 K min^{-1}.[3]

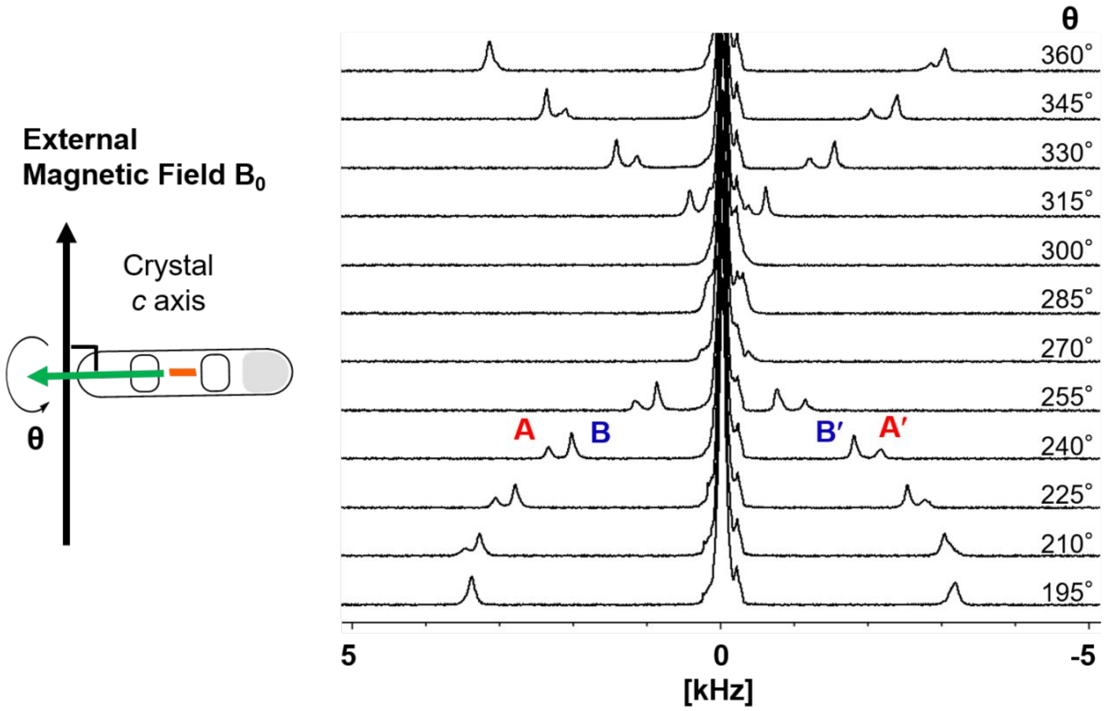

Figure S4. Solid-state ^{2}H-NMR spectra of a single crystal of **1′** at 296 K (θ = 195°–360°), with the long axis of the sample tube parallel to the *c* axis of the crystal (perpendicular to B_0).

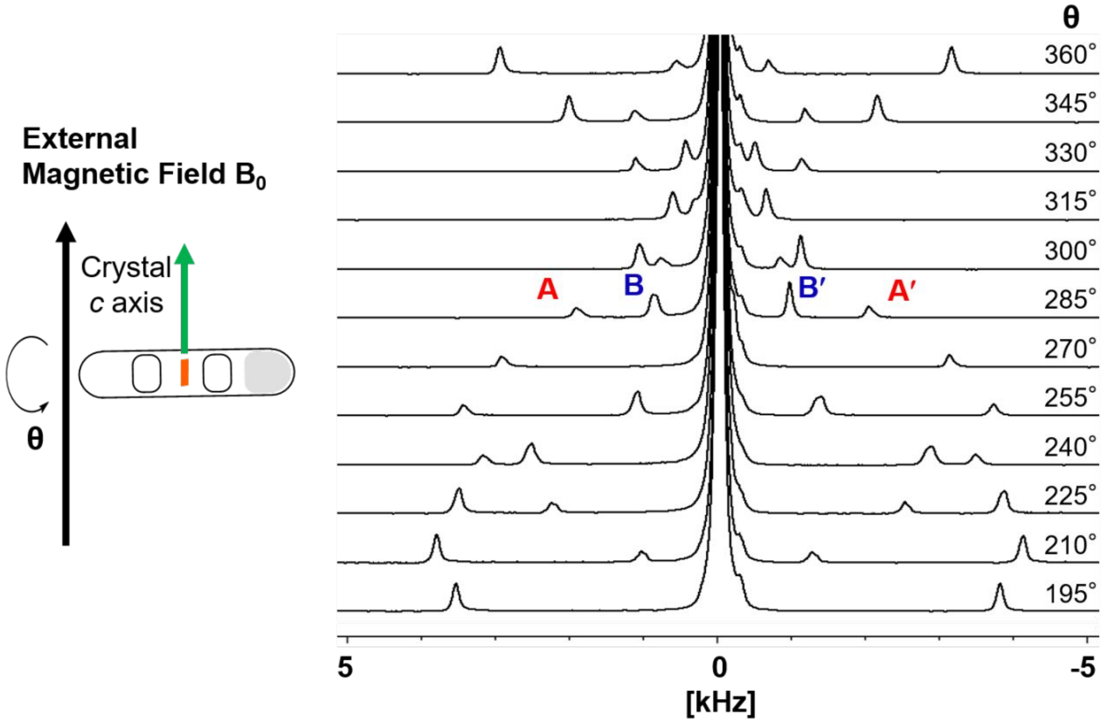

Figure S5. Solid-state ^{2}H-NMR spectra of single crystal **1′** at 296 K (θ = 195°–360°), with the long axis of the sample tube perpendicular to the *c* axis of the crystal (parallel to B_0).

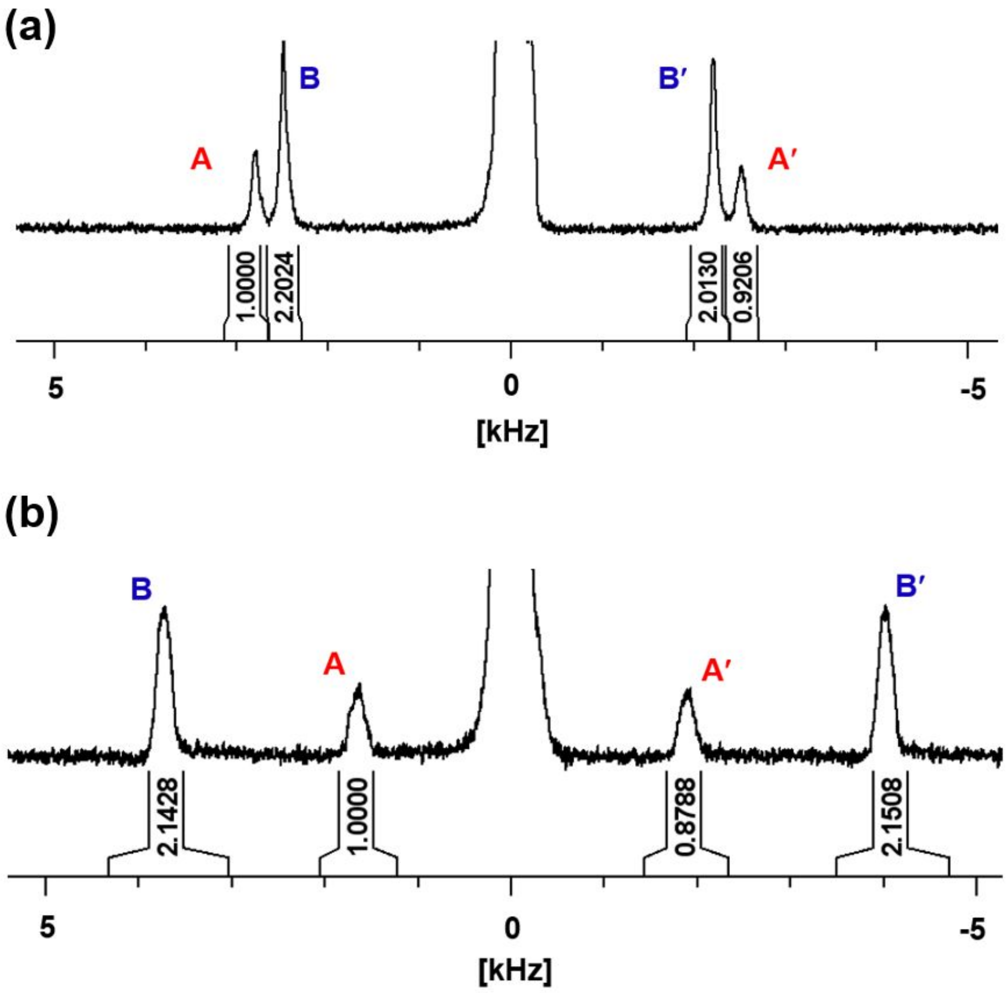

Figure S6. Integrals of peaks AA′ and BB′ of single crystal solid-state ^{2}H-NMR spectra at 296 K were measured for crystal **1′** in (a) parallel and (b) perpendicular samples. The integration ratio was A:B = 1:2.

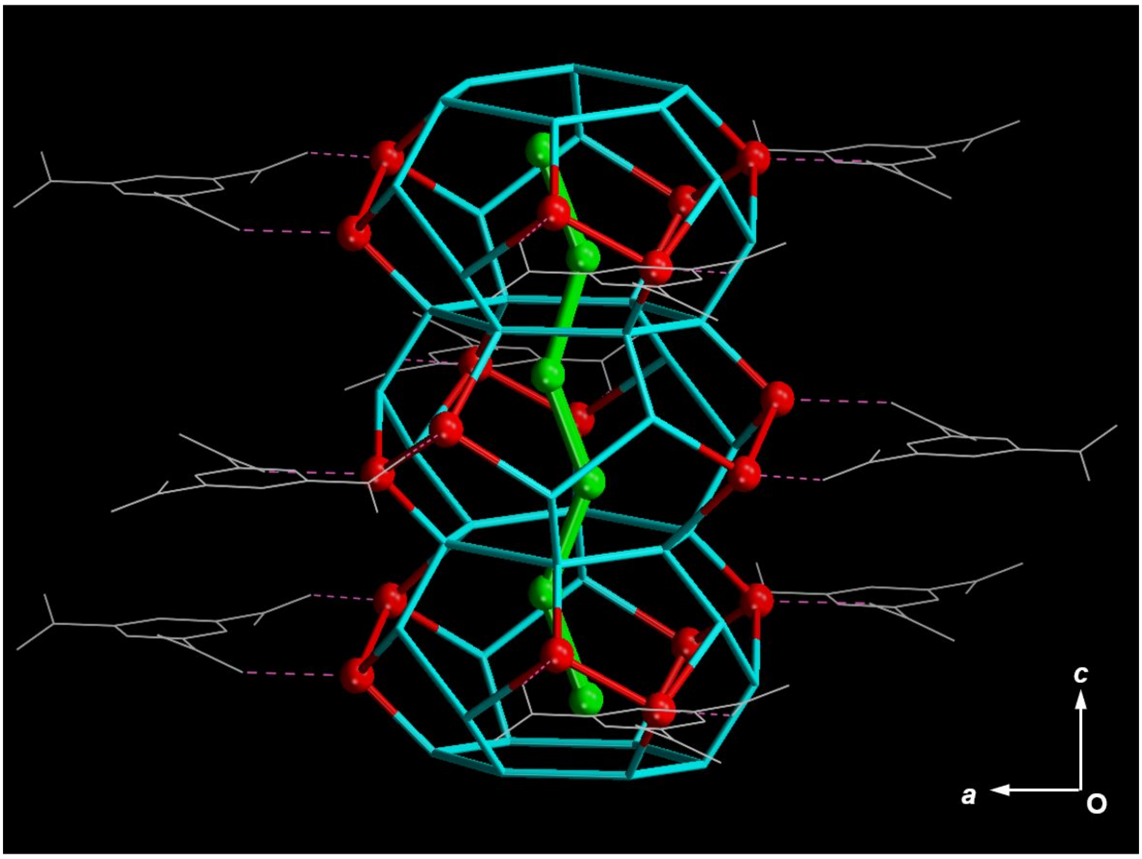

Figure S7. Structure of three units of WMCs and TMA^{3-} molecules (grey lines) in the porous framework in **2** at 253 K. The primary layers consist of six H_2O molecules H-bonded (pink dashed lines) to the O atoms of TMA^{3-} on the pore wall (red spheres) and six H_2O molecules not H-bonded to O atoms on the pore wall (blue lines), the secondary layers (blue lines) consist of six H_2O, and the tertiary regions (green spheres) consist of two H_2O molecules.

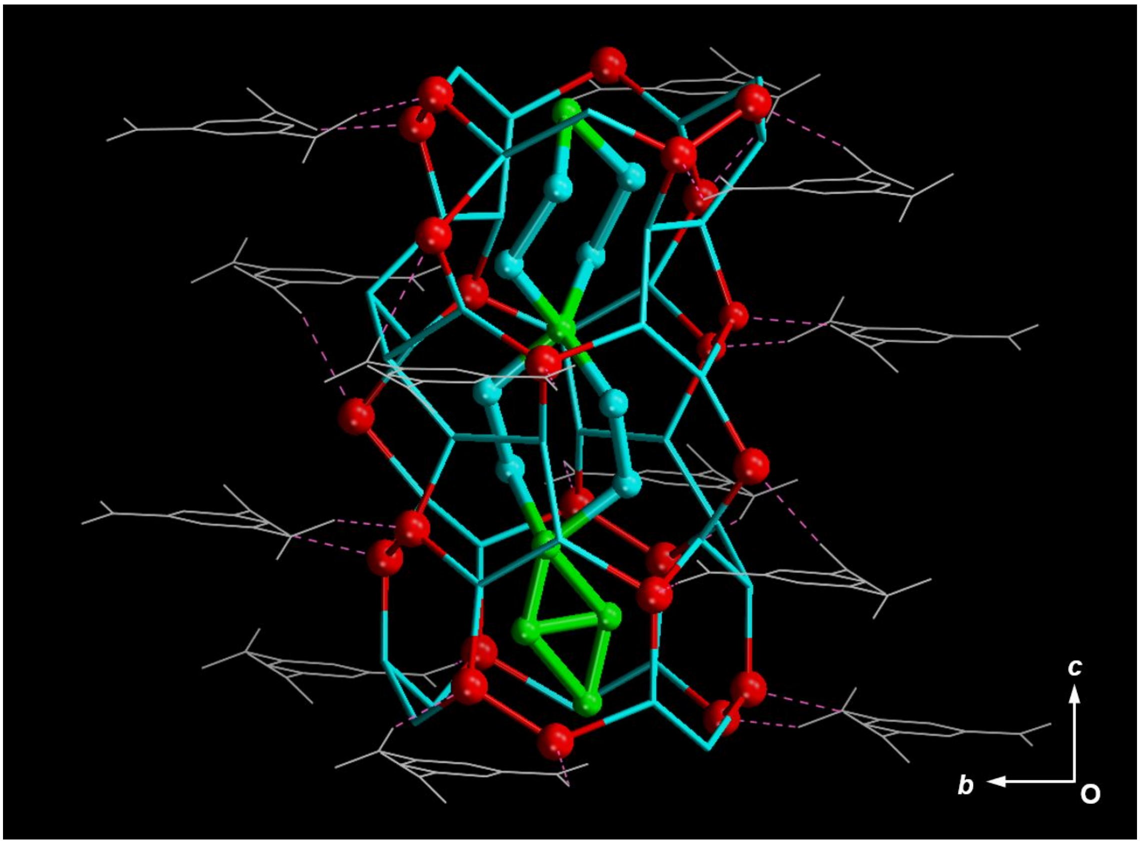

Figure S8. Unit structure of the premelting state and TMA^{3-} molecules (grey lines) in the porous framework in **1** at 198 K. The primary layer consists of 18 H_2O H-bonded to the O atoms of TMA^{3-} on the pore wall (red spheres) and 30 H_2O not H-bonded to O atoms on the pore wall (blue lines), the secondary layer (blue spheres) consists of eight H_2O, and the tertiary region (green spheres) consists of four H_2O molecules.

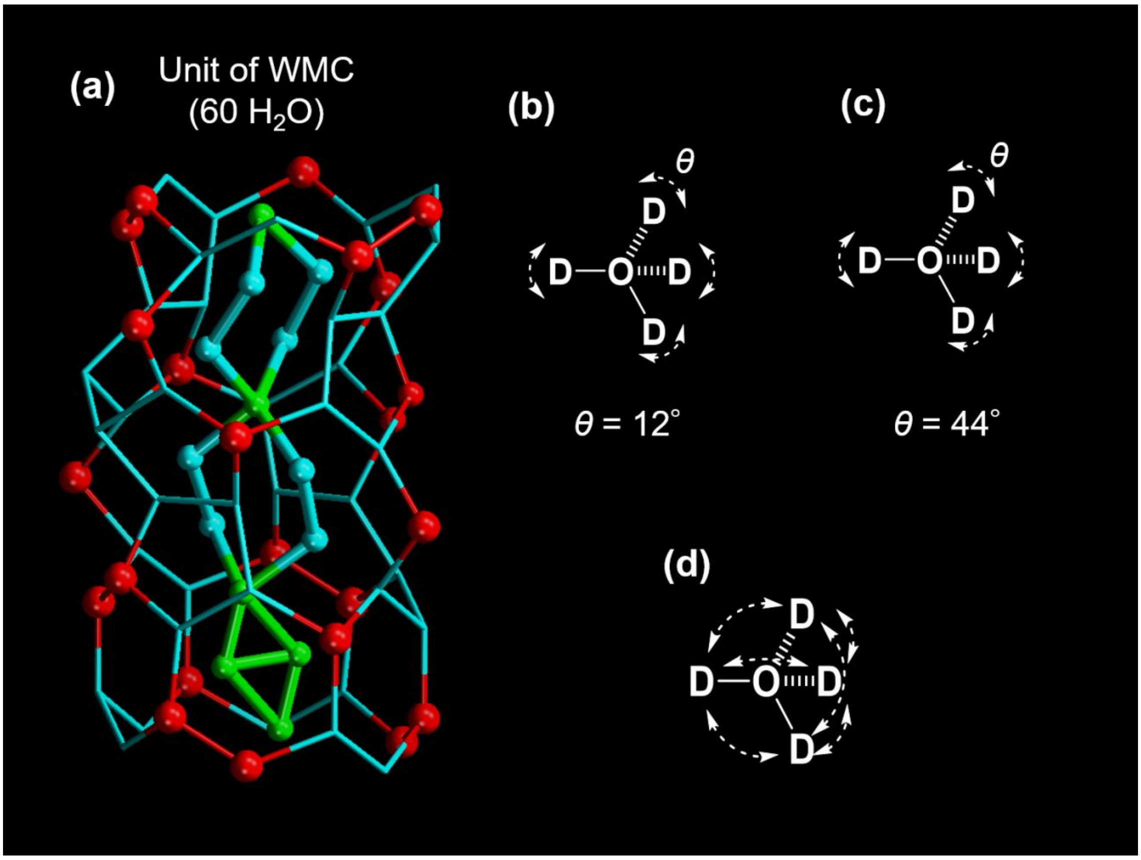

Figure S9. Motion modes used in the ^{2}H-NMR spectrum fitting of powder **1′** at 163 K based on the premelting WMCs structure derived from the X-ray structural analysis of **1** (a). D_2O in the primary layer H-bonded to O atoms of –COO^- groups (red spheres) exhibit rotational fluctuation at θ = 12 (b). D_2O in the primary layer not H-bonded to O atoms in the pore wall (blue line) and D_2O in the secondary layer (blue spheres) exhibit rotational fluctuation at θ = 44° (c). D_2O in the tertiary region (green spheres) exhibit isotropic rotation (d).

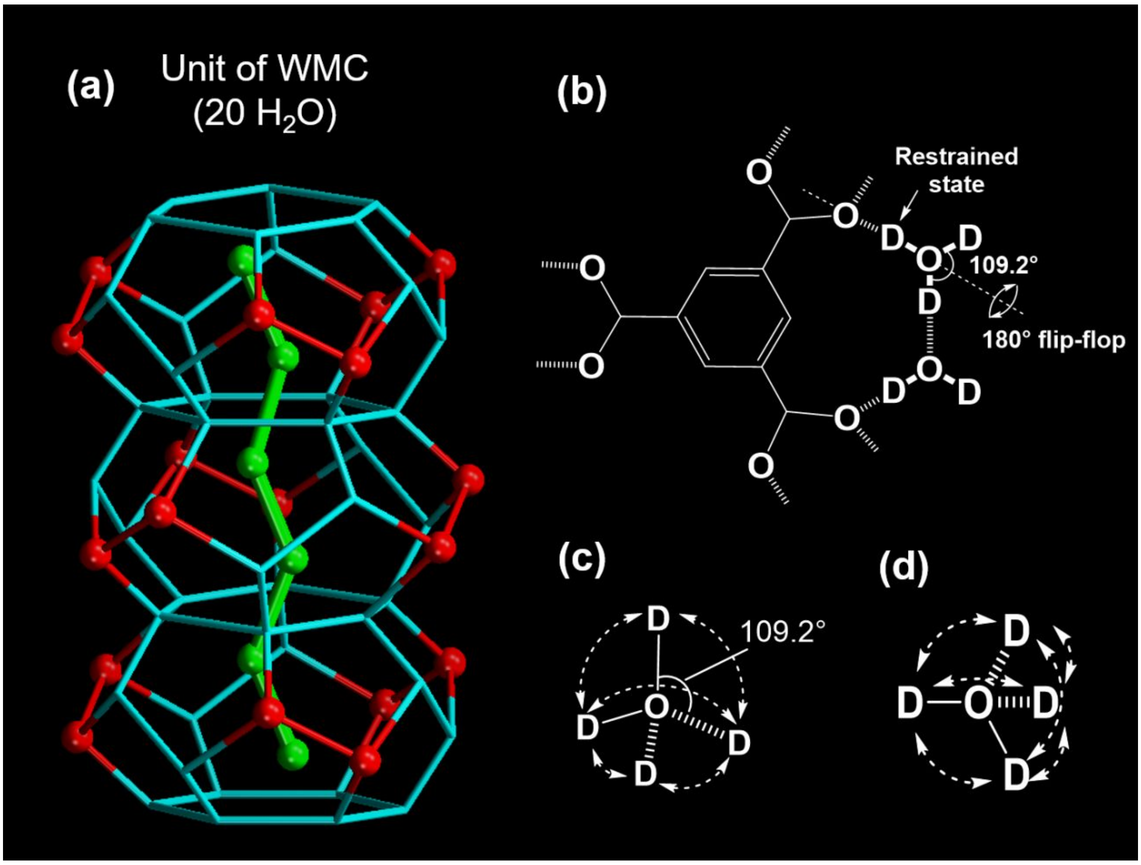

Figure S10. Motion modes used in the ^{2}H-NMR spectrum fitting of powder **1′** at 263 K based on the dynamic WMCs structure derived from the X-ray structural analysis of **2** (a). D_2O in the primary layers H-bonded to O atoms of –COO⁻ groups (red spheres) exhibit restraint by H-bonded to the O atoms of the carboxylate groups on the pore walls and 180° flip-flop motion around the hydrogen bond with the carboxyl group as the axis (b). D_2O in the primary layer not H-bonded to O atoms in the pore wall and D_2O in the secondary layers (blue line) exhibit a 4-site jump motion (c). D_2O in the tertiary regions (green spheres) exhibit isotropic rotation (d).

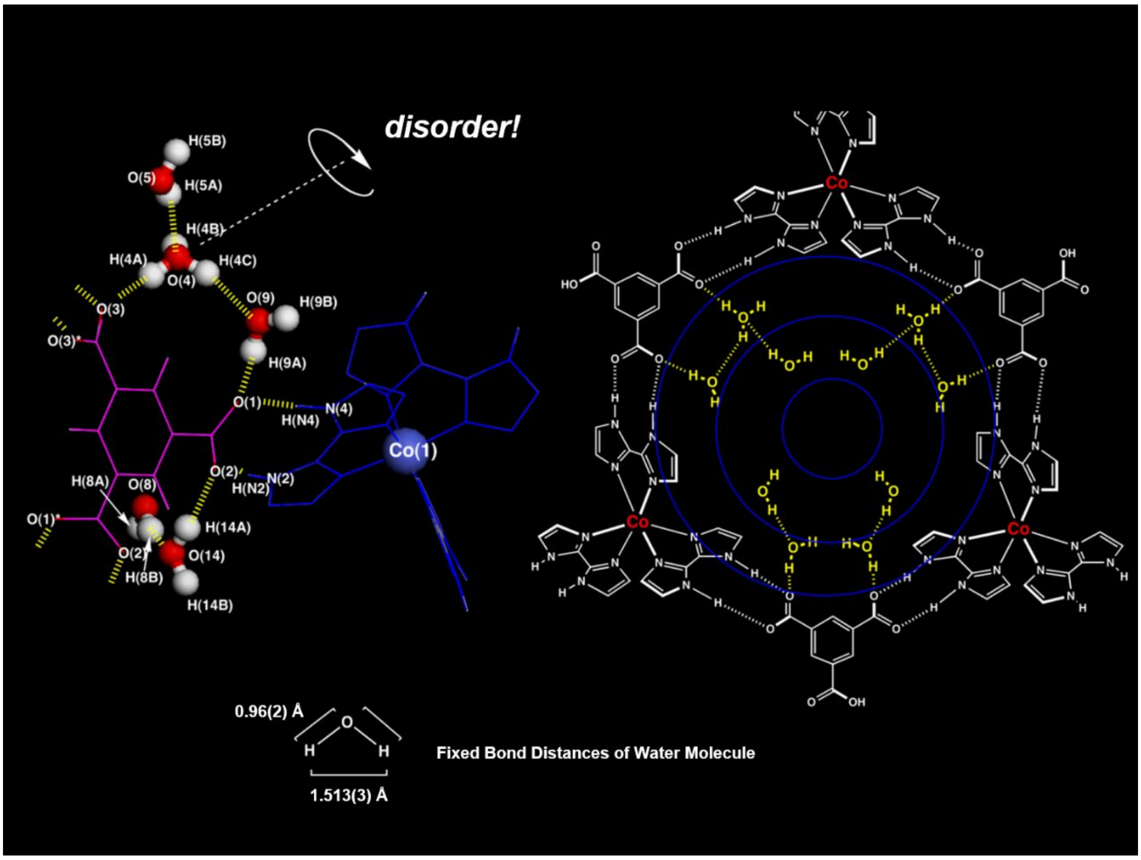

Figure S11. Neutron structural analysis of structural water in a water nanotube in **2**. H (4B) and H (4C) are disordered at 0.5 occupancy. Cyclic hydrogen bonds starting from the O atoms of trimesate and part of the secondary layer of water molecules were observed.

References

(1) Ohata, Y.; Kamebuchi, H.; Watanabe, K.; Kouchi, T.; Suzuki, Y.; Imaizumi, T.; Sugaya, T.; Mizuno, M.; Tadokoro, M. Slow Dynamics of Premelting Water Molecule. *Chemistry Select* **2019**, *4*, 6627–6633.

(2) Mizuno, M., Iwasaki, A., Umiyama, T., Ohashi, R. & Ida, T. Local Structure and Dynamics of Imidazole Molecules in Proton-Conducting Poly(vinylphosphonic acid)–Imidazole Composite Material. *Macromolecules* **2014**, *47*, 7469–7476.

(3) Tadokoro, M.; Ohhara, T.; Ohata, Y.; Suda, T.; Miyasato, Y.; Yamada, T.; Kikuchi, T.; Tanaka, I.; Kurihara, K.; Oguni, M.; Nakasuji, K.; Yamamuro, O.; Ryota, K. Anomalous Water Molecules and Mechanistic Effects of Water Nanotube Clusters Confined to Molecular Porous Crystals. *J. Phys. Chem. B* **2010**, *114*, 2091–2099.
